# Bloodstream infection by *Fusarium Solani* Complex in a paediatric patient affected by acute lymphoblastic leukaemia and P450 cytochrome polymorphism: A case report

**DOI:** 10.22034/cmm.2025.345277.1568

**Published:** 2026-02-01

**Authors:** Francesco De Leonardis, Vittorio Greco Miani, Vittoriana De Laurentiis, Michele Mastria, Mariachiara Servedio, Enza Pentassuglia, Roberta Koronica, Angela Vinella, Maria Addolorata Mariggiò, Nicola Santoro

**Affiliations:** 1 Division of Paediatric Haematology-Oncology, University of Bari, P.zza Giulio Cesare 11, 70124 Bari, Italy; 2 UOC Microbiology and Virology, Azienda Ospedaliera-Universitaria Policlinico of Bari, 70124 Bari, Italy; 3 Department of Biomedical Sciences and Human Oncology, University of Bari. Piazza Giulio Cesare 11, Bari 70124, Italy

**Keywords:** ALL, *Fusarium solani*, Invasive Fungal Infection, Pediatric

## Abstract

**Background and Purpose:**

*Fusarium* species are causative agents of a wide range of opportunistic infections, ranging from superficial to disseminated forms, particularly in nosocomial settings. Children with haematological malignancies may develop severe fungal infections, such as fusariosis, that have proven notoriously challenging to diagnose and treat, with high mortality rates.

**Case Presentation:**

This report aimed to describe an invasive *Fusarium* bloodstream infection in an eleven-year-old male patient treated for high-risk acute lymphoblastic leukaemia. The patient presented with neutropenic fever and necrotic papular skin lesions. *Fusarium solani* complex was identified in blood cultures. Initial empirical therapy with voriconazole led to partial improvement, but therapeutic drug monitoring showed persistently low plasma levels. Genetic analysis revealed CYP2C19*17 polymorphism, classifying the patient as an ultrarapid metabolizer. Treatment was switched to liposomal amphotericin B, resulting in clinical recovery.

**Conclusion:**

This case highlighted the importance of early diagnosis, targeted antifungal treatment, therapeutic drug monitoring, and pharmacogenetic testing in pediatric *Fusarium* infections.

## Introduction

*Fusarium* is a ubiquitous saprophytic hyaline fungus commonly isolated from plants, soil, insects, and water. Once considered only pathogenic in vegetables, it was not until 1973 that Cho et al. documented the first case of *Fusarium* bloodstream infection in a child with acute myeloid leukemia [1]. Ever since, opportunistic infections caused by *Fusarium* spp. have been increasingly described, especially in immunocompromised patients [2]. *Fusarium* species produce an infection belonging to the group of hyalohyphomycoses, characterized by the presence of hyaline, septate hyphae in tissues.

Recent molecular advances have led to the reclassification of the *Fusarium* genus into multiple species complexes. The *Fusarium solani* species *complex* (FSSC) includes over 60 phylogenetically distinct but morphologically similar species. Clinically, the most frequently encountered pathogenic groups are the FSSC and *Fusarium oxysporum* species complex, with *F. solani* and *F. oxysporum* responsible for over 50% and 20% of severe cases, followed by *F. verticillioides* and *F. proliferatum* [3–4].

In immunocompetent individuals, symptoms typically manifest as mild conditions, such as onychomycosis, keratitis, or allergies [3-5]. Conversely, immunocompromised patients have a greater risk of developing invasive and widespread infections, which often lead to high mortality rates. Fusariosis is more commonly manifested with pulmonary infections and peritonitis. Disseminated skin involvement is usually associated with fungemia. Given the relatively low incidence of fusariosis, there is a lack of clinical pediatric data, and many of the pediatric recommendations are extrapolated from adult series [6].

## Case Presentation

An eleven-year-old male affected by high-risk acute lymphoblastic leukaemia (ALL) and treated according to AIEOP-BFM ALL 2017 protocol, was hospitalised for 5 days due to neutropenic fever. Routine microbiological work-up and blood cultures were repeatedly carried out, and an empiric broad-spectrum treatment with cefepime and fluconazole was started. One week from the start of the fever, maculo-papules appeared on the upper and lower limbs. The rash progressively worsened with scattered violet-brown papules, many of them showing central umbilicated necrosis and peripheral swelling (Figure 1). Eight days from the onset of fever, the presence of spores and hyphae suggestive of a fungal infection was detected in a blood culture, and the mould was subsequently identified as belonging to the *F. solani* complex species (Figure 2).

**Figure 1:**
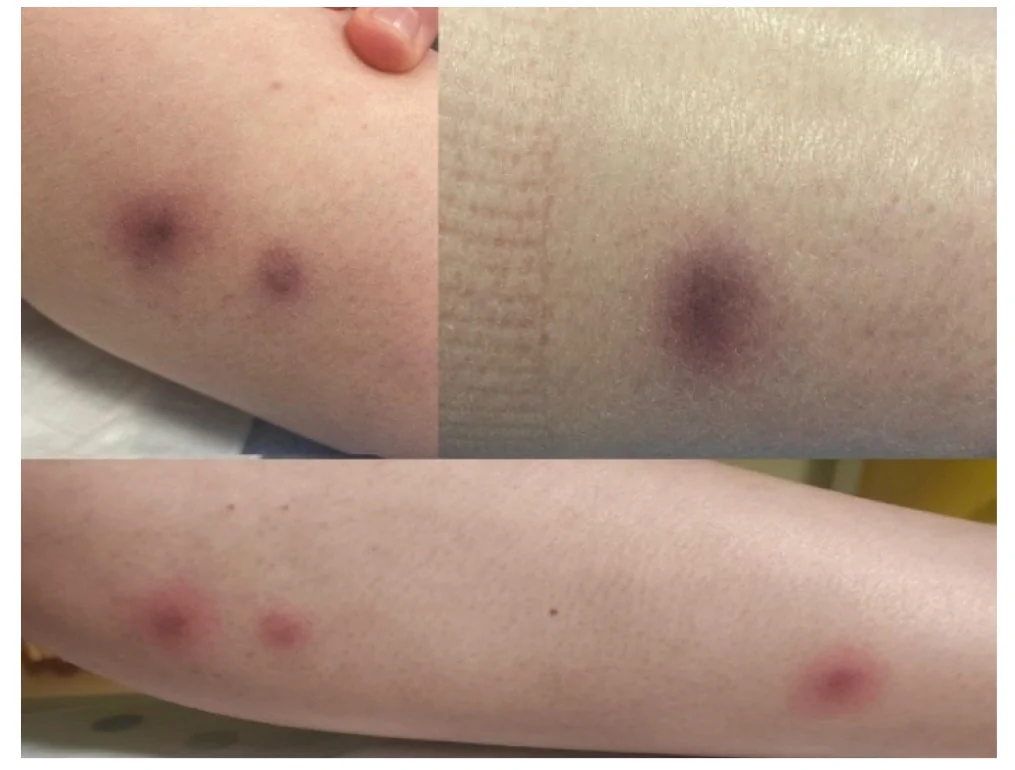
Multiple erythematous to violaceous papular and nodular skin lesions are visible on the upper and lower limbs of the patient. The lesions appeared well-circumscribed, some with central umbilication and surrounding induration, consistent with cutaneous manifestations of disseminated *Fusarium* infection.

**Figure 2:**
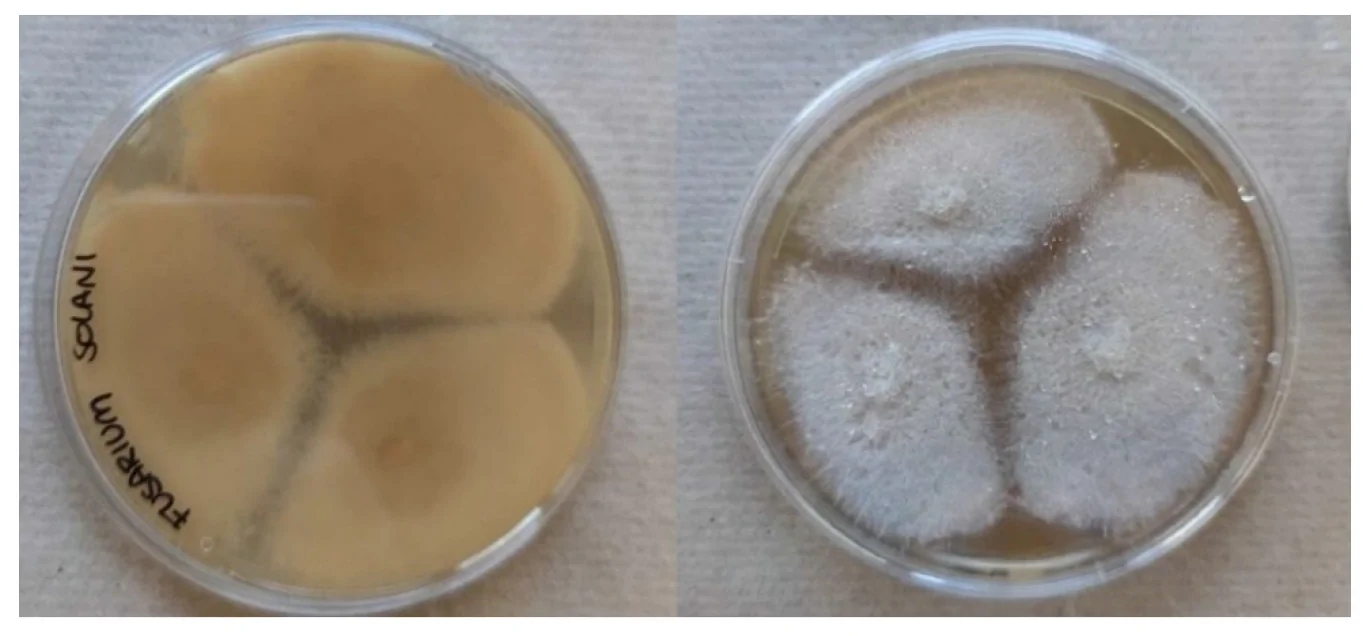
The image shows a culture of *Fusarium solani* grown on sabouraud agar medium. These colonies exhibit a cotton-like texture and are characterized by a coloration ranging from white to cream, typical of this species in culture after 24 h at 37 °C. The growth pattern displays rapid radial expansion from the center of inoculation, indicating active development of the aerial mycelium. Microbial identification by matrix-assisted laser desorption ionization–time of flight mass spectrometry was performed by transferring biomass from a single colony onto a dedicated steel target plate using a sterile swab. The procedure was carried out under a Class II biological safety cabinet to maintain aseptic conditions.

The peripherally inserted central venous catheter was promptly removed, the antibiotic was stopped, and empirical antifungal treatment was switched to IV voriconazole (6 mg/kg bid loading doses, followed by 4 mg/kg bid as maintenance dose). Extensive imaging and specialist evaluations ruled out additional fungal localizations. Five days from the start of IV voriconazole, clinical conditions improved, and inflammation markers decreased. Voriconazole was switched to the oral formulation, and therapeutic drug monitoring (TDM) was scheduled in an outpatient service.

Over the following 3 weeks, voriconazole TDM exhibited considerable variability and remained below the target range despite a progressive increase in the dose up to 13 mg/kg/die (Table 1). Genetic polymorphism analysis of cytochrome P450 was carried out, and the patient was found to be an “ultrarapid metabolizer” since the polymorphic variant CYP2C19*17 (-806C>T) was identified. Cytochrome polymorphism detection was performed by the PGX-CYP2C19 StripAssay® (GmbH). The DNA extraction from 5 ml EDTA whole blood samples was performed on the fully automated system ELITeInGenius^TM^ (ELITechGroup S.p.A., Italy), using Magtration® Technology. The allelic loci of the tested samples were studied through the CollectorTM sheet. Three different staining patterns resulted from wild-type, heterozygous polymorphic, and homozygous polymorphic loci. Finally, the metabolic features were associated with the resulting genotype. On the basis of the pharmacological data, voriconazole was replaced by 2.5 mg/kg/dose twice-weekly liposomal amphotericin B (LAmB). Complete disappearance of lesions occurred in 2 months. The LAmB prophylaxis lasted 5 months, and it was interrupted when the patient moved to the maintenance chemotherapy phase. No relevant side effects, signs, or symptoms of fusariosis reactivation have been noted since.

**Table 1:** Drug levels, total milligrams of drug administered, and dosage per kilogram during voriconazole therapy.

Day	Voriconazole Dose	Single Dose/Kg	Therapeutic Drug Monitoring
+4	200mg × 2	4 mg/Kg	1.8 ug/ml
+6	260mg × 2	5.2 mg/kg	3.5 ug/ml
+10	260mg × 2	5.2 mg/kg	7.5 ug/ml
+14	200mg × 2	4 mg/Kg	3.25 ug/ml
+18	200mg × 2	4 mg/Kg	1.88 ug/ml
+23	220mg × 2	4.4 mg/Kg	1.57 ug/ml
+26	240mg × 2	4.8 mg/Kg	0.97 ug/ml
+30	260mg × 2	5.2 mg/Kg	3.28 ug/ml
+33	260mg × 2	5.2 mg/Kg	3.22 ug/ml
+37	260mg × 2	5.2 mg/Kg	2.68 ug/ml
+39	260mg × 2	5.2 mg/Kg	2.29 ug/ml
+43	260mg × 2	5.2 mg/Kg	2.21 ug/ml
+47	200mg × 2	4 mg/Kg	3.95 ug/ml
+53	200mg × 2	4 mg/Kg	1.4 ug/ml
+57	260mg × 2	5.2 mg/Kg	1.03 ug/ml
+59	280mg × 2	5.6 mg/Kg	1.22 ug/ml
+63	300mg × 2	6 mg/Kg	0.72 ug/ml
+66	200mg × 2	4 mg/Kg	0.54 ug/ml
+70	210mg × 2	4.2 mg/Kg	0.82 ug/ml
+74	240mg × 2	4.8 mg/Kg	1.76 ug/ml
+79	240mg × 2	4.8 mg/Kg	1.3 ug/ml
+87	260mg × 2	5.2 mg/Kg	0.85 ug/ml
+94	280mg × 2	5.6 mg/Kg	1.13 ug/ml
+101	300mg × 2	6 mg/Kg	0.77 ug/ml
+103	300mg × 2	6 mg/Kg	0.49 ug/ml
+108	320mg × 2	6.4 mg/Kg	0.9 ug/ml
+115	320mg × 2	6.4 mg/Kg	0.58 ug/ml

## Discussion

*Fusarium* spp. is the third most common mould causing infections after *Aspergillus* and *Mucorales*. The majority of adult infections occur in tropical and subtropical regions where these fungi are endemic [4]. Conversely, the highest number of pediatric fusariosis cases is reported in Europe, and hospital environments, particularly plumbing and water systems, can act as reservoirs for infection [7-8]. Entry into the body may occur through inhalation, ingestion, or traumatic inoculation, although in many cases, including the present case, the source remains unidentified [9-10]. Prolonged and profound neutropenia, often induced by chemotherapy and corticosteroid use, represents a major risk factor for invasive fusariosis [11-12]. In both children and adults, the most frequent pattern of disseminated fusariosis is the same as observed in the present patient, which is the combination of cutaneous lesions and positive blood cultures [13]. Skin lesions usually present numerous nodular areas of erythema with tenderness, darkening, and central pustular necrosis. They usually evolve, spreading widely over the body, and may coexist at different stages of evolution [13-14].

Fungemia is a common manifestation of invasive fusariosis. In a recent pediatric review, it was reported significantly more frequently in patients younger than 3 years [8]. Pulmonary involvement is less common in pediatric patients (18%), compared to adults (50%). Conversely, osteoarticular involvement is more frequent in children, maybe owing to differences in skeletal structure and vascularisation [15]. Anecdotal infection of the heart, eye/central nervous system, kidney, and/or liver/spleen is described in the pediatric population [7].

Culture and histology of infected tissue or fluid remain the cornerstone of diagnosis, while molecular techniques have been currently considered the best diagnostic option [3-16]. Since blood cultures yield positive results in only 40% of invasive cases, direct examination of tissue, particularly through skin biopsy, allows a rapid and more accurate diagnosis. Histopathological findings include acute branching septate hyaline hyphae, sometimes with sporulation. The genus *Fusarium* produces filamentous colonies, but microscopically, phialides produce mono- and pluriseptate spindle-shaped conidia; nevertheless, species identification may be challenging [3].

In contrast to most filamentous fungi, *Fusarium* species have a peculiar ability to cause fungemia. This characteristic differentiates *Fusarium* infections from those caused by other molds, such as *Aspergillus spp.*, which are typically tissue-invasive but rarely bloodborne [4, 14]. Imaging plays a crucial role in the detection of invasive fungal infections and the assessment of the extent of disease; however, treatment decisions should not be based on imaging results alone [16]. Other diagnostic tools, such as galactomannan and β-1,3-D-glucan assays, may show cross-reactivity with *Fusarium* spp., but lack specificity and pediatric validation, and are only marginally recommended [3,16].

Clinical management of disseminated fungal diseases deserves a prompt diagnosis and a targeted treatment, but no randomized trials have yet assessed the effectiveness of different antifungal treatments for *Fusarium* species [17]. The largest study available is a multicenter retrospective analysis of 236 patients, both children and adults, diagnosed between 1985 and 2011 across 44 centers in 11 countries [12]. The aforementioned study reported a 90-day survival rate of 27% for patients treated with amphotericin B deoxycholate, 53% for those treated with voriconazole, and 48% for those treated with LamB [12]. The effectiveness of combination therapy with voriconazole and LAmB was comparable to that of monotherapy [13]. Current guidelines recommend voriconazole or LAmB as first-line treatment [16], while posaconazole is reserved for salvage therapy since it has shown a 48% success rate in small case series [16-19]. Amphotericin B deoxycholate is no longer recommended, while combination therapy as initial treatment is especially important for critically ill patients [16-17]. According to the 2020 European Conference on Infections in Leukemia guidelines, rare fungal infections, like fusariosis, in pediatric cancer patients should be treated following the same recommendations as those for adults [19]. A recent pediatric review involving 106 patients reported a 38% overall mortality rate (40/106) [7] with combination therapy (amphotericin B and voriconazole) reducing the mortality to 37% (10/27), compared to 48% with amphotericin B deoxycholate monotherapy (10/21) and 21% with voriconazole alone (6/28) [8]. No clear correlation has been described between in vitro voriconazole susceptibility or high minimum inhibitory concentration (MIC) values and patient outcomes [7-15-20].

A case series of 233 patients demonstrated an increased use of voriconazole as primary treatment, particularly as monotherapy, achieving a notable 60% 90-day survival rate [12]; despite typically high MICs for *Fusarium* species [21]. The TDM of voriconazole has proven crucial for optimizing treatment of *Aspergillus* infections, owing to the extreme inter- and intra-individual pharmacokinetic variability, as also observed in the present case (Table 1) [22]. TDM ensures adequate drug exposure, enhances clinical outcomes, and minimizes adverse drug reactions [23]. Current guidelines recommend targeting serum levels of 2–6 µg/mL, extrapolated from *Aspergillus* data, as a reasonable approach for *Fusarium* [23].

According to the 2020 guidelines, if voriconazole serum concentrations remain subtherapeutic despite two appropriate dose adjustments, it is recommended to consider switching to an alternative antifungal agent and conducting CYP2C19 genotype testing (moderate recommendation, level III evidence); similarly, antifungal agents other than voriconazole should be considered for patients known to be CYP2C19 ultra-rapid metabolizers (strong recommendation, level III evidence) [23].

Apart from antifungal treatment, the optimal care for patients with fusariosis involves surgical debridement of infected tissues, removal of venous catheters in cases of confirmed catheter-related fusariosis, and restoration of immune function [3-16]. In neutropenic patients, granulocyte colony-stimulating factors or transfusions may help shorten neutropenia duration [13-16]. In the present case, despite escalating voriconazole dosing, plasma levels remained persistently subtherapeutic in a way that it was replaced with LAmB; subsequently, the CYP2C19*17 (-806C>T) polymorphism explained the persistently ineffective plasma concentrations of voriconazole.

## Conclusion

This experience confirms that a high degree of suspicion and early diagnosis are warranted for assuring a favorable prognosis in invasive *Fusarium* infection. This report emphasizes the role of voriconazole dose optimization by TDM, eventually integrated by CYP2C19 genotyping, as a crucial tool in children, too, for a potential prompt drug switch in case of treatment inefficacy or unexpected toxicities.
